# lncRNA-LET Regulates Glycolysis and Glutamine Decomposition of Esophageal Squamous Cell Carcinoma Through miR-93-5p/miR-106b-5p/SOCS4

**DOI:** 10.3389/fonc.2022.897751

**Published:** 2022-05-10

**Authors:** Xincheng Su, Cong Xue, Chengke Xie, Xianzhe Si, Jie Xu, Wenbo Huang, Zhijun Huang, Jianqing Lin, Zhiyao Chen

**Affiliations:** ^1^ Department of Gastrointestinal and Esophageal Surgery, The 2nd Affiliated Hospital of Fujian Medical University, Quanzhou, China; ^2^ Department of Oncology, The 2nd Affiliated Hospital of Fujian Medical University, Quanzhou, China

**Keywords:** esophageal squamous cell carcinoma, lncRNA-LET, miR-93-5p, miR-106b-5p, SOCS4

## Abstract

**Background:**

Dysregulated non-coding RNAs exhibit critical functions in various cancers. Nonetheless, the levels and corresponding functions of cirCSNX14 in esophageal squamous cell carcinoma (ESCC) yet remain to be elucidated.

**Methods:**

Initially, the aberrant low levels of lncRNA-LET within ESCC tissues are validated *via* qRT-PCR observations. Moreover, the effects of lncRNA-LET upregulation on cell proliferation *in vitro* are determined. In addition, a series of assays determining the mechanistic views related to metabolism is conducted. Furthermore, the effects of lncRNA-LET in affecting tumor growth are investigated *in vivo* in a mouse model. Moreover, the interactions between lncRNA-LET and its networks are predicted and determined by RNA immunoprecipitation-assisted qRT-PCR as well as luciferase reporter assays.

**Results:**

The downregulation of lncRNA-LET is correlated to the poor prognosis of ESCC patients. Moreover, the upregulated expression of lncRNA-LET could have reduced the cell viability. *In vivo* tumor inhibition efficacy assays showed that an increase of lncRNA-LET presented excellent inhibitory effects on cancer proliferation as reflected by tumor weight and volume in mice. Finally, the mechanistic views regarding the effects of miR-106b-5p or miR-93-5p and SOCS4 on ESCC are related to the feedback of lncRNA-LET.

**Conclusion:**

Collectively, this study suggested that lncRNA-LET miR-93-5p or the miR-106b-5p–SOCS4 axis may provide great potential in establishing ESCC therapy.

## Introduction

Esophageal squamous cell carcinoma (ESCC), a primary esophageal cancer subtype, has remained as one of the significant healthcare challenges globally (1). Despite several advancements in treatment options and the development of various medical technologies, the treatment against ESCC remains highly challenging due to its location, which significantly hampers early prognosis and ease of surgical sectioning (2). Moreover, understanding the underlying mechanism is another highly challenging task in developing therapeutics against this malignant disease (3).

In addition to various attributes of increased glucose uptake and lactate production, the intrinsically available long non-coding ribose nucleic acids (lncRNAs) offer various essential functions in cancer prognosis (4, 5). Interestingly, several reports indicated the association of lncRNAs with cancer cell survival and proliferation through cell metabolic activities (6). In a case, lncRNA UCA1 enhanced tumorigenesis in bladder cancer through the upregulation of HK2, a key enzyme for metabolism (7). In another case, the downregulation of lncRNA DUXAP8 markedly inhibited the expression of HK2 and LDHA and thus decreased the glucose uptake in NSCLC cells (8).

Similarly, abnormal lncRNA levels have been identified in ESCC, which could assist in understanding the origin and proliferation of cancer cells towards early prognosis and the development of therapeutic options—for instance, it was reported that lncRNA POU3F3 could be beneficial for the early screening of ESCC (9). Moreover, lncRNA HCG22 could prevent the migration of cells in ESCC (8). In addition, lncRNA-uc002yug.2 could improve the RUNX1 combination with MALAT1, PEG10, and CASC9, showing an association with ESCC (10). These findings, based on lncRNA levels, would undoubtedly offer great potential in the early diagnosis or prognosis of ESCC. However, the critical and targeted functionalities of lncRNA-LET in ESCC cells still remain unclear. Furthermore, abnormally expressed miRNAs have been reported within various cancers (11), for instance, miR-185-5p serves as a tumor-promoting gene (11), and KLF3 plays critical biological impacts on cancer apoptosis (12).

In general, lncRNAs serve as the spongers of microRNAs (miRNAs) that are abnormally expressed in various tumors (13, 14), affecting other target genes *via* base pairing (15),—for instance, it was reported that the miR-548k in ESCC exerted oncogenic functions through downregulating the lncRNA-LET expression (16). On the contrary, upregulation of miR-93-5p and miR-106b-5p within esophageal carcinoma has been reported in the literature (17, 18). In another instance, the suppressors of cytokine signaling (SOCS-1 and SOCS-3) were revealed to be implicated in ESCC progression (19). Moreover, the association of lncRNA TUSC7, miR-616, and SOCS4 has been revealed in endometrial carcinoma (16, 20). Notably, miR-1290 was abnormal in lung adenocarcinoma, contributing to cancer progression through targeting SOCS4 (21). Inspired by these facts, herein we intend to detect the levels and functions of lncRNA-LET and its network in ESCC cancers. Moreover, the underlying regulated gene and protein expressions were also investigated to thoroughly understand the function of lncRNA-LET.

## Experimental Section

### Specimen Collection, Cell Culture, and Transfection

In total, 80 ESCC and matched non-carcinoma samples were altogether collected in cases from the 2nd Affiliated Hospital of Fujian Medical University (FMU). The patients who underwent chemotherapy or radiotherapy were excluded from our work to avoid affecting the gene expression. Each case had provided informed consent. Our study protocols gained approval from the medical ethics committee of the 2nd Affiliated Hospital of FMU.

The ESCC cells (KYSE0, TE-1, KYSE150, KYSE410, and Eca-109) were provided by the American Type Culture Collection (Manassas, VA, USA) and cultured within Dulbecco’s modified Eagle’s medium (Gibco, Grand Island, NB, USA) containing 8% or 10% fetal bovine serum (Gibco) and 1% penicillin-streptomycin (Gibco).

The complementary deoxyribose nucleic acid (cDNA) plasmids of lncRNA-LET were purchased from Genomeditech Co, Ltd. (Shanghai, China). Puromycin (2 μg/ml, Solarbio Co., Ltd., Beijing, China) was used to select stable transfection cell lines. The miR-106b-5p mimics, miR-93-5p, and corresponding controls were provided by GeneChem Co., Ltd. (Shanghai, China), which were transfected into cells with Lipofectamine 2000 (Invitrogen, NY, USA). The pcDNA-lncRNA-LET and controls were provided by GenePharma (Shanghai, China) and transfected using Lipofectamine 2000. Then, the pcDNA-(SOCS4) vectors were designed and synthesized by Biovector Science Inc. (Beijing, China):

lncRNA-LET WT: 5′-UACUUUGCCAAAUAGCACUUUA-3′
lncRNA-LET MUT: 5′-UACCCAGCUAGGCCCACGUAGA-3′
hsa-miR-93-5p: 5′-GAUGGACGUGCUUGUCGUGAAAC-3′
hsa-miR-106b-5p: 5′-UAGACGUGACAGUCGUGAAAU-3′
SOCS4 WT: 5′-AGAAGUAGACAAUUGCCACUUUU-3′
SOCS4 MUT: 5′-AGAAGUAGACAAUUGCAGCGACU-3′

### Cell Proliferation

The cell counting kit (CCK)-8 (Solarbio, Beijing, China) was adopted for examining cell proliferation. Briefly, non-treated or treated cells (5 × 10^3^/well) were inoculated into 96-well plates for incubation. Each well was added with CCK-8 solution (10 μl) for another 2 h of incubation. Finally, the plate was scanned using a multiple-microplate reader (Bio-rad, Shanghai, China) at 450 nm.

### RNA Extraction and qRT-PCR Assay

Initially, RNAstorm™ Kit (#CD501, Biotium, Fremont, USA) was adopted for extracting total RNA, which was later prepared into cDNA with miRNA Reverse Transcription Kit from Qiagen (Hilden, Germany). Next, cDNA amplification was performed by 7500 Fast Real-Time System (Bio-Rad) after mixing with SYBR Green PCR Master Mix. The relative RNA expression was obtained with the standard 2^−ΔΔCt^ method, in which β-actin and U6 were applied as the endogenous references. The primer sequences are as follows:

- β-Actin
  F: 5′-AGCTCTGTAACCACAGGTTC-3′
  R: 5′-GGGCGGTTGTTGGTCACAGA-3′
- U6
- F: 5′-GCGCGCAACGGCGACTCA-3′
  R: 5′-GAGGTAGGCGCTCCAGACGA-3′
- hsa-miR-93-5p
  F: 5′-GCCGTTAAAGTCGTGTTC-3′
  R: 5′-CAGAGCAGGGATCGATCTA-3′
- hsa-miR-106b-5p
- F: 5′-TCCCGACAAACGAGCTTTGA-3′
  R: 5′-AGGCAATGATCAGCGAATTC-3′
- SOCS4
- F: 5′-GGGCACGGACAGCATGTTGC-3′
  R: 5′-CCGTGAGTTAATGCTGCCTGGG-3′

### Dual-Luciferase Reporter Assay

We obtained lncRNA-LET and miRNA targets from the sRNA target dataBase (https://www.hsls.pitt.edu/obrc/index.php?page=URL20110217163843). From the starBase analyses, the binding sites of miR-106b-5p and miR-93-5p within the non-coding region of the SOCS4 gene were observed. Primarily, the mutant (SOCS4 MUT) and wild type (WT) of SOCS4 were prepared *via* Genomeditech Co., Ltd. (Shanghai, China), which were later inserted in the respective vector. Then, miR-106b-5p and miR-93-5p or control mimics were transfected into cells and then treated with pRL-SV40 (Promega). Finally, an analytical assay exploring the luciferase activities was conducted with the luciferase reporter assay kit (Solarbio).

### RNA Immunoprecipitation–qRT-PCR

RNA Immunoprecipitation Kit (Sigma, St. Louis, USA) was adopted for RNA immunoprecipitation (RIP) assay. Initially, cell lysates were obtained using the abovementioned RNA extraction kit and subjected to centrifugation. The magnetic beads pre-treated with anti-IgG (ab1-470, Abcam, Cambridge, UK) or anti-AGO2 (ab2-81, Abcam) were incubated with the obtained supernatant. Subsequently, qRT-PCR was employed to determine the enrichment of lncRNA-LET, miR-93-5p, and miR-106b-5p.

### Western Blotting

Initially, RIP assay lysis buffer was used to obtain total protein from cellular lysates, and then the protein was quantified with the Bradford method. Subsequently, the total denatured protein was scattered with 10 or 12% SDS-PAGE and then transferred onto nitrocellulose membranes (Sigma). Then, 5% bovine serum albumin (BSA) was used to block the membranes and incubated using anti-SOCS4, anti-HK2, anti-LDHA, anti-amino acid transporter-2 (ASCT2), and anti-glutaminase 1 (GLS1) primary antibodies, respectively. β-Actin was applied as a loading control. Then, horseradish peroxidase (HRP)-labeled anti-rabbit IgG antibody (#7074, CST, USA) or HRP-labeled anti-mouse IgG antibody (#7076, CST, USA) was adopted for incubating the membranes. The blots were visualized by enhanced chemiluminescence substrates. All reagents, except antibodies, are obtained from Beyotime (Shanghai, China).

### Determination of Glucose Uptake, Lactate, ATP Production, Glutamine, and α-KG

Initially, controls or lncRNA-LET-overexpressed plasmids were utilized to transfect the ESCC cells for a 48-h period. Furthermore, the corresponding colorimetric assay kits (BioVision, CA, USA) were used to determine the glucose and lactate concentrations. The ATP levels in each group were detected by an ATP colorimetric/fluorometric assay kit (Sigma). The contents of glutamine and α-KG were detected with a glutamine/glutamate determination kit (Sigma). Finally, similar detections were conducted after the transfection with miRNA mimics.

### *In Vivo* Investigations

The *in vivo* investigations using mice were conducted strictly following the Institutional Animal Care and Use Committee guidelines from the 2nd Affiliated Hospital of FMU. The animals were raised under a 12-h light/dark cycle room in an SPF environment. To establish the tumor model, BALB/c nude mice (age, 6 weeks old) were given an injection of Eca-109 cells transfected with normal vector or lncRNA-LET pcDNA vector (200 μl of 1 × 10^6^ cells/mouse) on the dorsal side of the animal. Furthermore, the tumor volume was calculated using the following equation: volume = (length × width^2^)/2, in which length indicates the longest dimension, while width represents the shortest dimension of the tumor. Finally, the mice were euthanized with CO_2_ inhalation, and the excised tumors were weighed accurately.

### Immunohistochemistry Assay

Initially, the excised tumor tissues from nude mice were prepared as paraffin slides, followed by antigen retrieval. Furthermore, 5% BSA was adopted for blocking the tissue sections for a 1-h period, followed by incubation using antibodies against anti-Ki67, SOCS4, HK2, LDHA, ASCT2, and GLS1, respectively (Cell Signaling Technology, Danvers, MA, USA). Finally, the slides were observed using a microscope.

### Statistical Analysis

The experimental results are displayed as mean ± SD. The statistical analysis was performed, using GraphPad Prism 9.0, through one-way ANOVA or Student’s *t*-test plus Tukey’s *post-hoc* test at a defined statistical significance of *P* <0.05 (*P < 0.05, **P < 0.01, and ***P < 0.001). Associations among lncRNA-LET and clinical features were analyzed through chi-square test. The correlation between lncRNA-LET miR-93-5p/miR-106b-5p and SOCS4 was examined by Pearson’s correlation test.

## Results

### lncRNA-LET Profiles in ESCC Tissues

**Figure 1:**
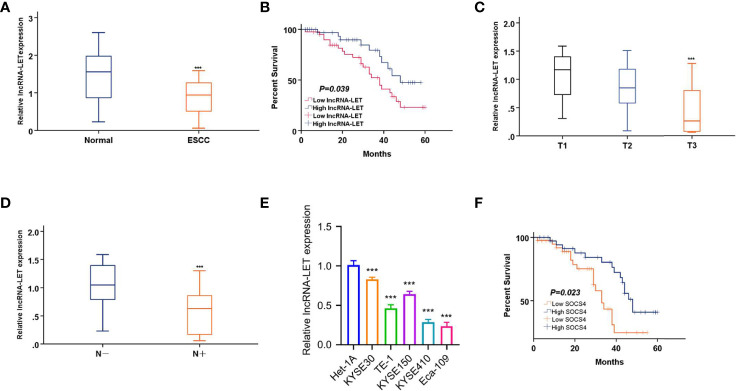
**(A)** lncRNA-LET expression within esophageal squamous cell carcinoma (ESCC) and matched non-carcinoma samples was measured by qRT-PCR. **(B)** The overall survival (OS) rate in cases showing lncRNA-LET downregulation (*n* = 40) and lncRNA-LET upregulation (*n* = 40) was analyzed by the Kaplan–Meier plot. **(C)** lncRNA-LET expression in different clinical T stages was measured by qRT-PCR. **(D)** lncRNA-LET expression within clinical LN-negative and LN-positive was measured by qRT-PCR. **(E)** lncRNA-LET expression in ESCC cell lines and the normal cell het-1a was measured by qRT-PCR. **(F)** The OS rate in cases showing SOCS4 downregulation (*n* = 40) and SOCS4 upregulation (*n* = 40) was analyzed by the Kaplan–Meier plot. ***P < 0.001.

Initially, cancerous and paracancerous ESCC tissues were analyzed by qRT-PCR to explore the clinical functionality of lncRNA-LET. It was observed that lncRNA-LET was expressed significantly lower (*P* < 0.001) in ESCC tissues compared to the paracancerous tissues (**Figure 1A**). Then, the relationship between lncRNA-LET and the prognosis of ESCC was established by the overall survival rate evaluation (**Figure 1B**). The overall survival time in patients with low levels of lncRNA-LET indicated poor prognosis, demonstrating the anti-cancer potential of lncRNA-LET. The lncRNA-LET decreased with an increase of clinical T stage in ESCC patients (*P* < 0.001) (**Figure 1C**), and lncRNA-LET in clinical LN-negative was expressed significantly higher than that in clinical LN-positive (*P* < 0.001) (**Figure 1D**). Owing to a wide variety of cells in the tumor microenvironment, several other cell lines were selected to more precisely characterize the expression levels of lncRNA-LET in cancer cells. As depicted in **Figure 1E**, lncRNA-LET was downregulated within the ESCC cell lines compared to the paracancerous samples, indicating the anti-cancer efficacy of lncRNA-LET. Furthermore, the overall survival time in patients with high levels of SOCS4 indicated good prognosis (**Figure 1F**).

Taking the median expression value of lncRNA-LET in the 80 cases of ESCC (**Figure 1A**) as a cutoff value, the patients were randomly distributed into two groups as low and high lncRNA-LET expression groups (*n* = 40). Based on the relationship of lncRNA-LET with clinical features, the lncRNA-LET levels were firmly related to the tumor size and differentiation, TNM stage, and lymph node metastasis. However, it should be noted that the obtained results were independent of the age and gender of the patients (**Table 1**).

**Table 1:** The relationship between lncRNA-LET and the clinical features of patients.

Clinical characteristics	High lncRNA-LET (*n* = 40)	Low lncRNA-LET (*n* = 40)	*P*-value
Age			0.3705
≥60 years	21	17	
<60 years	19	23	
Gender			0.1432
Female	15	9	
Male	25	31	
Tumor size			0.0389
≤3 cm	29	20	
>3 cm	11	20	
Differentiation degree			0.0452
High/moderate	33	25	
Low	7	15	
Lymph node metastasis			0.0322
Positive	35	27	
Negative	5	13	
Clinical stage			0.0125
I–II	22	11	
III–IV	18	29	

### Influence of lncRNA-LET on Proliferation, Glycolysis, and Glutamine Decomposition of ESCC Cells

**Figure 2:**
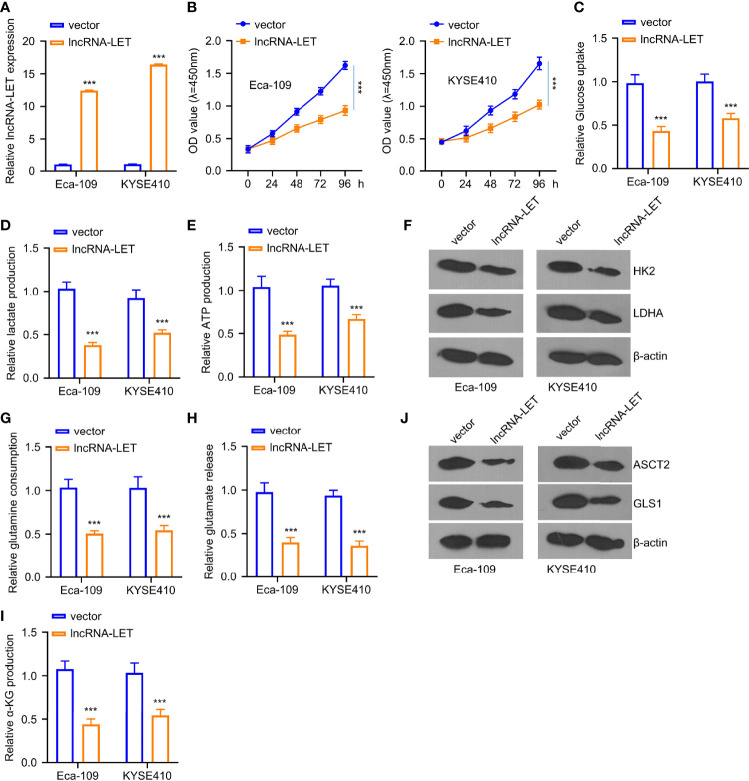
**(A)** The transfection efficiency of plasmid pcDNA lncRNA-LET was determined through qRT-PCR. **(B)** Cell viabilities in Eca-109 and KYSE410 cells transfected with or without lncRNA-LET pcDNA at 0, 24, 48, 72, and 96 h were measured through CCK-8 assay. **(C–E)** The levels of glucose, lactate, and ATP production were detected by the glycolysis kit. Western blotting was used to detect **(F)** the expressions of HK2 and ldhA proteins in Eca-109 and KYSE410 cells with or without lncRNA-LET transfection. **(J)** ASCT2 and gls1 protein levels in different groups (vector, lncRNA-LET). **(G–I)** The levels of glutamine, glutamic acid, and α-glutamine in cells after control vector or lncRNA-LET pcDNA transfection were detected by α-KG detection kit. ***P < 0.001.

Based on the results displayed in **Figure 1C**, two kinds of ESCC cells, *i*.*e*., Eca-109 and KYSE410, were selected due to the extremely low expression of lncRNA-LET. As depicted in **Figure 2A**, lncRNA-LET plasmid pcDNA overexpression could significantly increase the expression of lncRNA-LET in Eca-109 and KYSE410 lines. The transfection would substantially inhibit the viabilities of Eca-109 and KYSE410 cells (**Figure 2B**). Owing to the engagement of lncRNAs in the metabolism of cancer cells *via* regulating transporters and enzymes (7), in this study, the glucose content, lactate generation, and ATP after the transfection of the lncRNA-LET plasmid were evaluated. It was observed that the overexpression of lncRNA-LET presented an apparent inhibition of glucose intake, ATP generation, and lactate generation within both cell lines (**Figures 2C–E**). Moreover, two crucial enzymes (HK2 and LDHA) for glycolysis were markedly decreased with the overexpression of lncRNA-LET (**Figure 2F**). Collectively, we confirm that the upregulation of lncRNA-LET had significantly repressed the glycolysis process intracellularly. Furthermore, the glutamine, α-glutamine, and glutamic acid levels in Eca-109 and KYSE410 cells were determined by an α-ketoglutarate (α-KG) detection kit. Glutamate, glutamine, and α-KG within cells transfected with the lncRNA-LET plasmid were significantly decreased (**Figures 2G–I**), suggesting the inhibition of lncRNA-LET on metabolism. In addition, the expression levels of GLS1 and ASCT2 protein related to glutamine uptake and metabolism were decreased with the overexpression of lncRNA-LET in both cell lines (**Figure 2J**) (22, 23).

### lncRNA-LET Sponge miR-106b-5p and miR-93-5p

**Figure 3:**
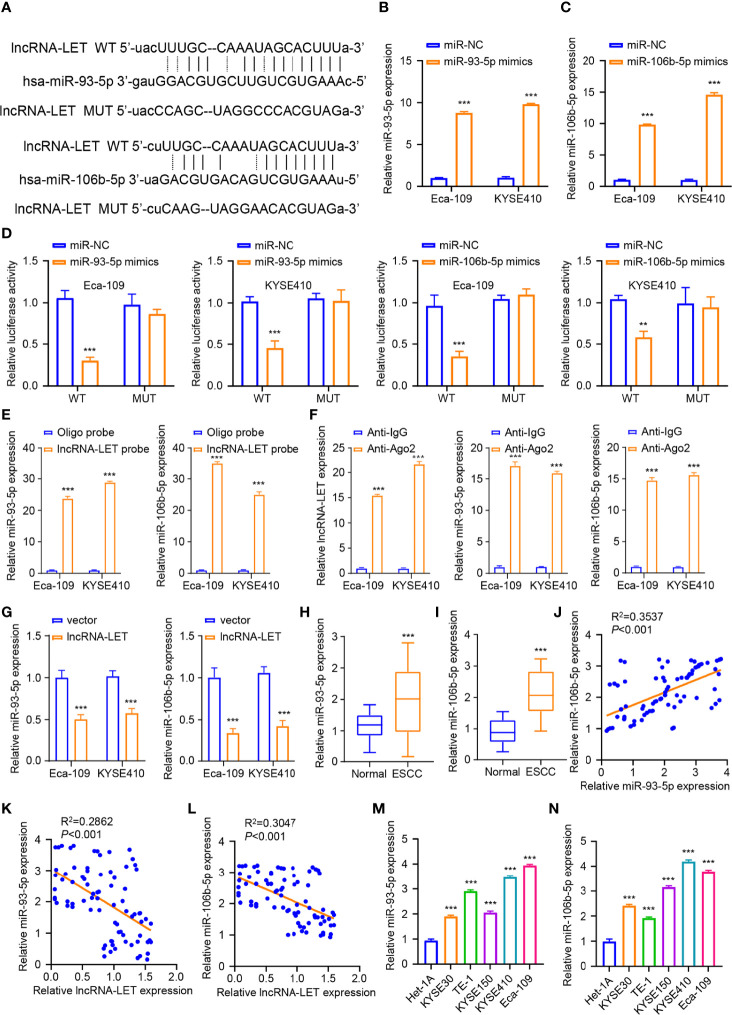
**(A)** The binding sites for miR-106b-5p and miR-93-5p predicted on lncRNA-LET were checked by starBase database. We conducted qRT-PCR to measure the **(B)** miR-93-5p levels after miR-nc mimic or miR-93-5p mimic transfection. **(C)** The miR-106b-5p level with miR-93-5p mimic or miR-nc mimic transfection. **(D)** Wild-type lncRNA-LET plasmid or binding site mutant plasmid luciferase activity affected by miR-106b-5p or miR-93-5p mimic transfection was detected by dual-luciferase activities. **(E)** The interaction of lncRNA-LET and with miR-93-5p/miR-106b-5p was verified through RNA pull-down assay. **(F)** The enrichment of lncRNA-LET, miR-93-5p, and miR-106b-5p was performed through RNA immunoprecipitation–qRT-PCR. **(G)** The miR-93-5p and miR-106b-5p levels within Eca-109 and KYSE410 cells after lncRNA-LET transfection were measured through qRT-PCR. We conducted qRT-PCR for detecting **(H, I)** miR-106b-5p and miR-93-5p expression within clinical tissue samples. **(L)** The miR-93-5p level and **(M)** miR-106b-5p level within esophageal squamous cell carcinoma (ESCC) cell and healthy esophageal epithelial cell line het-1a. **(J)** The correlation between the relative expressions of miR-106b-5p and miR-93-5p in the patient samples. **(K, L)** The association of lncRNA-LET with miR-93-5p/miR-106b-5p expressions. **(M, N)** The expressions of miR-93-5p and miR-106b-5p within ESCC cells (KYSE150, KYSE410, Kyse 0, TE-1, and Eca-109). *P < 0.05, **P < 0.01, and ***P < 0.001.

The starBase database was employed to analyze the miRNA binding site of lncRNA-LET (**Figure 3A**) to elucidate the aspects of targeted miRNA. It was observed that the lncRNA-LET wild type could bind to miR-106b-5p and miR-93-5p. Eca-109 and KYSE410 cells were subjected to miR-93-5p mimic treatment or non-treatment to confirm the prediction accuracy. To explore these aspects, miRNA expression was determined through qRT-PCR. From **Figure 3B**, miR-93-5p mimic transfection could remarkably increase the expression of miR-93-5p (*P* < 0.001) in both selected cell lines. Similarly, miR-106b-5p mimic transfection markedly increased miR-106b-5p expression compared to miR-nc in both selected cell lines (*P* < 0.001, **Figure 3C**). Then, the luciferase reporter gene experiment was performed in Eca-109 and KYSE410 cells. In comparison to miR-nc, the overexpressed miR-93-5p or miR-106b-5p could have significantly decreased the wild-type lncRNA-LET vector luciferase activity in Eca-109 and KYSE410 cells. Contrarily, the inhibition was retracted with the mutation of the predicted binding sites of miR-106b-5p or miR-93-5p (**Figure 3D**). Subsequently, we carried out the RNA pull-down assay by the biotin-labeled lncRNA-LET probe. As depicted in **Figure 3E**, it was confirmed that lncRNA-LET cells directly sponge mir-106b-5p or miR-93-5p. Furthermore, the RIP–qRT-PCR assay observations presented that the Ago2 group enriched more lncRNA-LET and miR-106b-5p/miR-93-5p, suggesting an interaction of lncRNA-LET with miR-106b-5p/mir-93-5p (**Figure 3F**). To verify the oriented association of lncRNA-LET with miR-93-5p/miR-106b-5p, the miR-106b-5p and miR-93-5p levels in Eca-109 and KYSE410 cells after lncRNA-LET overexpression derived from the use of pcDNA were detected. Compared with a vector, the overexpression of lncRNA-LET substantially downregulated the miR-106b-5p and miR-93-5p expression (**Figure 3G**). Then, the miR-93-5p (**Figure 3H**) and miR-106b-5p (**Figure 3I**) expressions within 80 ESCC subjects and matched non-carcinoma samples were detected, in which their expression in ESCC was remarkably elevated within cancer tissues. Moreover, a correlation between the relative expressions of miR-93-5p and miR-106b-5p in the patient samples was established (**Figure 3J**). In addition, negative correlations between lncRNA-LET and miR-93-5p (**Figure 3K**) or (**Figure 3L**) expression were demonstrated by Pearson correlation coefficient analysis. Furthermore, the miR-93-5p (**Figure 3M**) and miR-106b-5p (**Figure 3N**) expressions were increased within the ESCC cell lines (KYSE150, KYSE410, Kyse 0, TE-1, and Eca-109).

### lncRNA-LET Targets miR-93-5p/miR-106b-5p to Regulate Glycolysis and Glutamine Decomposition in ESCC Cells

**Figure 4:**
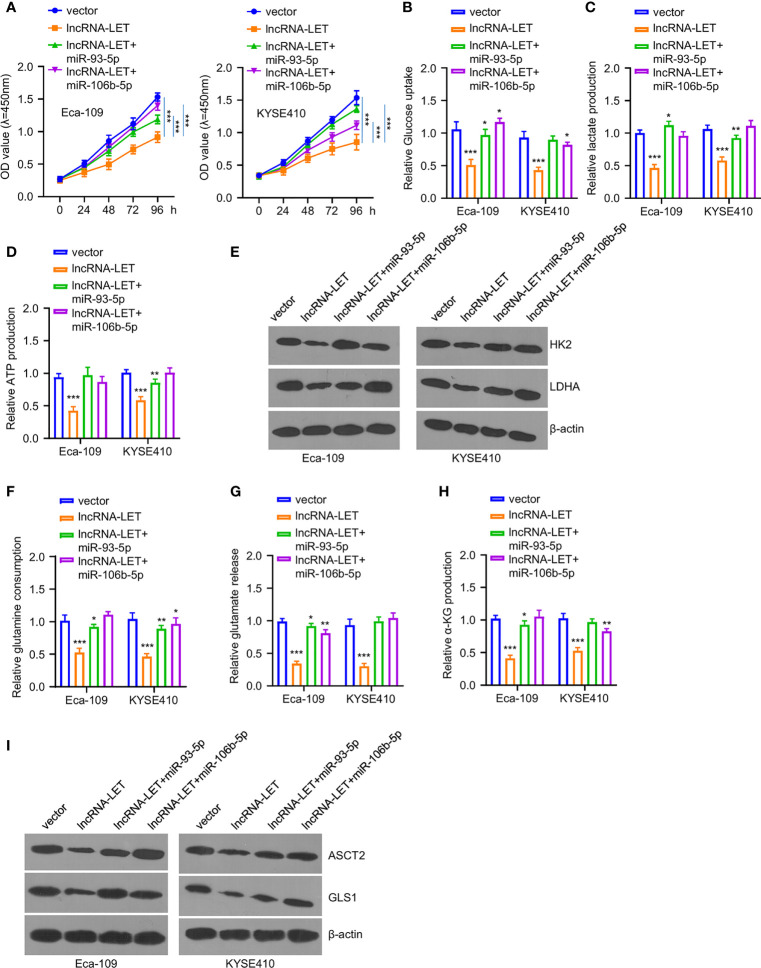
**(A)** Cell viability in cells after lncRNA-LET transfection or lncRNA-LET and miR-93-5p or miR-106b-5p transfection was measured with CCK-8 kit. **(B–D)** Glucose consumption, lactate production, and ATP generation within cells after lncRNA-LET transfection or lncRNA-LET and miR-93-5p or miR-106b-5p transfection were defined by a glycolysis kit. We performed Western blotting for detecting **(E)** hK2 and ldhA protein expression within cells after lncRNA-LET transfection or lncRNA-LET and miR-106b-5p or miR-93-5p transfection and **(I)** ASCT2 and gls1 protein levels within cells after lncRNA-LET transfection or lncRNA-LET and miR-106b-5p or miR-93-5p co-transfection. **(F–H)** The glutamine, glutamate, and α-KG levels within cells after lncRNA-LET transfection or lncRNA-LET and miR-106b-5p or miR-93-5p co-transfection were measured through α-KG detection kit. *P < 0.05, **P < 0.01, and ***P < 0.001.

To explore the effects of miR-93-5p or miR-106b-5p on lncRNA-LET-induced metabolism inhibition and tumor inhibition effects on ESCC cells, the cell viability of Eca-109 and KYSE410 cells was determined after treating with a vector, lncRNA-LET, and co-transfection (lncRNA-LET+miR-93-5p and lncRNA-LET+miR-106b-5p) (**Figure 4A**). Notably, it was observed that the cell proliferation ability in the selected cell lines was significantly reduced in the overexpression of the lncRNA-LET treatment group. In contrast, cell viability was increased after co-incubation of miR-106b-5p or miR-93-5p. In addition, the overexpression of lncRNA-LET inhibited the glucose contents, lactate generation, and ATP production intracellularly (**Figures 4B–D**). In contrast, the co-transfection of miR-106b-5p or miR-93-5p with lncRNA-LET partially increased the glucose contents, lactate generation, and ATP production, suggesting the tumor promotion roles of miR-106b-5p or miR-93-5p, along with their negative correlation with lncRNA-LET. The HK2 and ldhA protein levels in different groups of Eca-109 and KYSE410 cells revealed that the overexpression of lncRNA-LET had decreased the levels of HK2 and ldhA proteins in cells. Simultaneously, miR-106b-5p or miR-93-5p co-transfection partially recovered the HK2 and ldhA protein levels in ESCC cells (**Figure 4E**). As depicted in **Figures 4F–H**, the overexpressed lncRNA-LET inhibited glutamine, glutamate, and α-KG levels, while the co-incubation of miR-106b-5p or miR-93-5p partially abrogated the inhibition effect of lncRNA-LET. In contrast, the levels of ASCT2 and gls1 proteins in different groups of Eca-109 and KYSE410 cells showed different trends (**Figure 4I**). With the single transfection of lncRNA, the levels of ASCT2 and gls1 protein in cells were decreased, while the miR-106b-5p or miR-93-5p co-transfection partially increased the ASCT2 and gls1 protein levels in cells.

### Both miRNAs Target SOCS4

**Figure 5:**
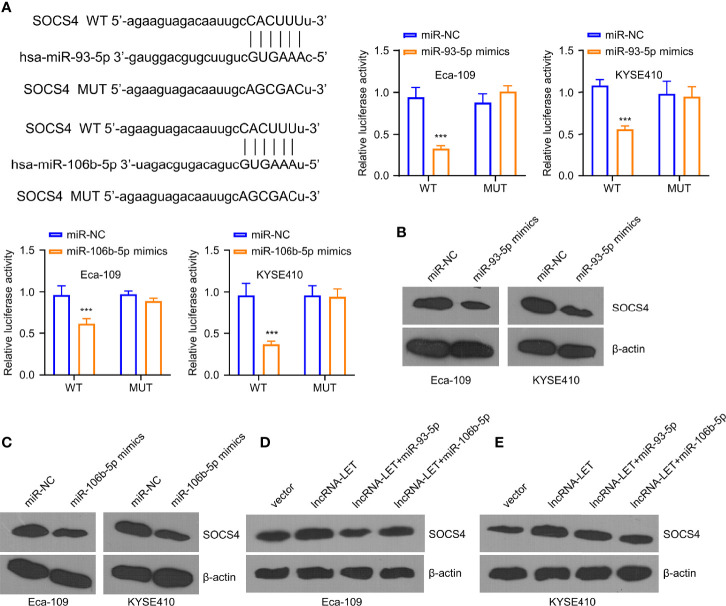
miR-93-5p/miR-106b-5p regulate the levels of SOCS4. **(A)** The predicted binding sites for SOCS4 gene on miR-93-5p and miR-106b-5p were analyzed by the starBase database. Western blotting was used to determine **(B, C)** SOCS4 protein in cells subjected to miR-93-5p or miR-106b-5p transfection and **(D, E)** in cells transfected with vector, lncRNA-LET, or lncRNA-LET+miR-106b-5p or miR-93-5p, respectively. *P < 0.05, **P < 0.01, and ***P < 0.001.

To investigate the downregulated miR-93-5p/miR-106b-5p expression, we analyzed their binding sites by starBase online database. It was observed from the experimental results that miR-106b-5p and miR-93-5p were associated with SOCS4. Furthermore, miR-106b-5p or miR-93-5p overexpression could inhibit SOCS4 in Eca-109 and KYSE410 cells (**Figure 5A**). However, no inhibition was observed in the SOCS4 mutant group. As shown in Western blotting, the expression of SOCS4 protein in Eca-109 and KYSE410 cells was decreased in the cases of pre-treatment by miR-106b-5p or miR-93-5p mimics (**Figures 5B, C**). Unlike the negative regulation of miR-106b-5p or miR-93-5p to SOCS4, lncRNA-LET overexpression increased the expression levels of SOCS4 protein, while less SOCS4 protein was detected in the co-transfection group (**Figures 5D, E**).

### The Effects of SOCS4 on the Inhibition of Glycolysis and Glutamine Decomposition

**Figure 6:**
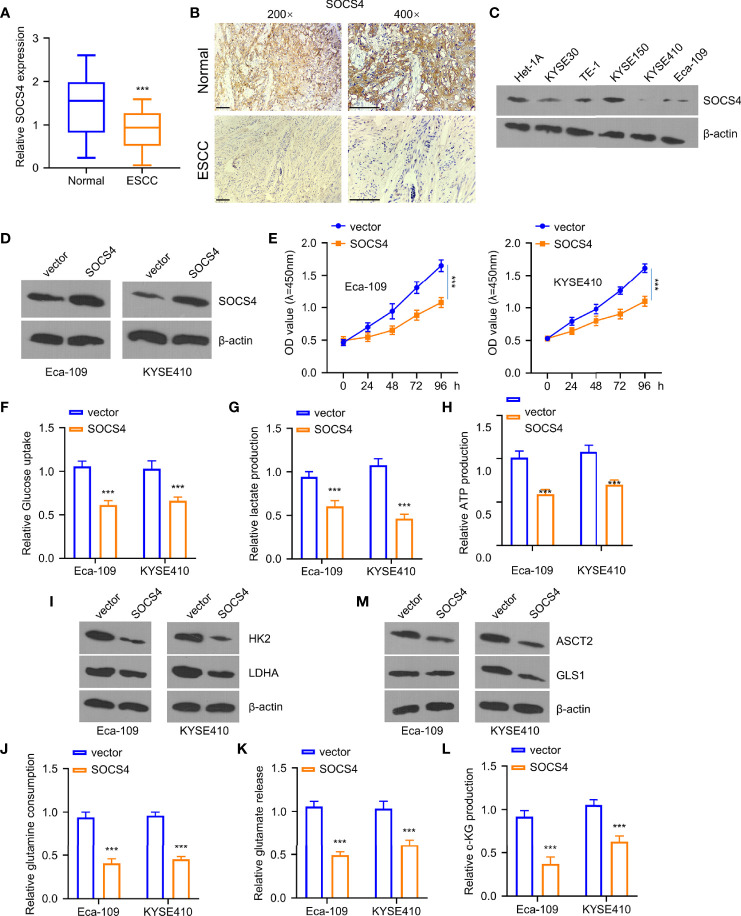
Overexpression of SOCS4 inhibited glycolysis and glutamine decomposition of esophageal squamous cell carcinoma (ESCC) cells. Western blotting (WB) **(A)** and immunohistochemistry **(B)** were used for determining SOCS4 expression within 80 ESCC and matched non-carcinoma samples. **(C)** SOCS4 protein levels within ESCC cells and healthy cells (het-1a) and **(D)** the overexpression of SOCS4 efficiency were detected by the WB method. **(E)** The cell viability of Eca-109 and KYSE410 cells at 0, 24, 48, 72, and 96 h was detected by the CCK-8 kit. **(F–H)** The glucose consumption, lactate production, and ATP in different groups (vector, SOCS4) of Eca109 and KYSE410 cells were defined by the glycolysis kit. **(I)** The levels of hK2 and ldhA protein in different groups (vector, SOCS4) of Eca-109 and KYSE410 cells and **(M)** gls1 and ASCT2 protein levels within cells with or without SOCS4 transfection. **(J–L)** Glutamate, glutamine, and α-KG expression in cells transfected with/without SOCS4 transfection was detected by α-KG detection kit. *P < 0.05, **P < 0.01, and ***P < 0.001.

Furthermore, the levels of SOCS4 in clinical samples were measured *via* qRT-PCR and immunohistochemistry to define the roles of SOCS4 in glycolysis and glutamine decomposition. As displayed in **Figures 6A, B**, the SOCS4 levels were dramatically reduced within ESCC tissues compared to the adjacent non-carcinoma samples. Furthermore, the SOCS4 protein expression levels were significantly lower within various ESCC cell lines of KYSE 0, TE-1, KYSE150, KYSE410, and Eca-109 than those of healthy human immortal esophageal epithelial cells (het-1a) (**Figure 6C**). Accordingly, Eca-109 and KYSE410 cell lines with the lowest SOCS4 expression levels were used to determine the roles of SOCS4. Compared with a vector, SOCS4 plasmid transfection can effectively overexpress the SOCS4 protein level in both cell lines (**Figure 6D**). Unlike the tumor-promoting roles of miR-106b-5p or miR-93-5p, SOCS4 overexpression dramatically inhibited cell proliferation (**Figure 6E**). Moreover, the regulatory role of SOCS4 in cell metabolism was similar to that of lncRNA-LET (**Figures 6F–H**). In addition, the overexpression of SOCS4 inhibited glucose uptake and lactate generation, together with ATP generation, while SOCS4 upregulation decreased the levels of HK2 and ldhA protein in the selected cells (**Figure 6I**). The obligatory increase of SOCS4 levels inhibited the glutamine, glutamate, and α-KG levels (**Figures 6J–L**) as well as resulted in the reduction of the expression levels of ASCT2 and gls1 proteins in various ESCC cells (**Figure 6M**).

### lncRNA-LET Upregulation Inhibited ESCC Cell Proliferation *In Vivo*

**Figure 7:**
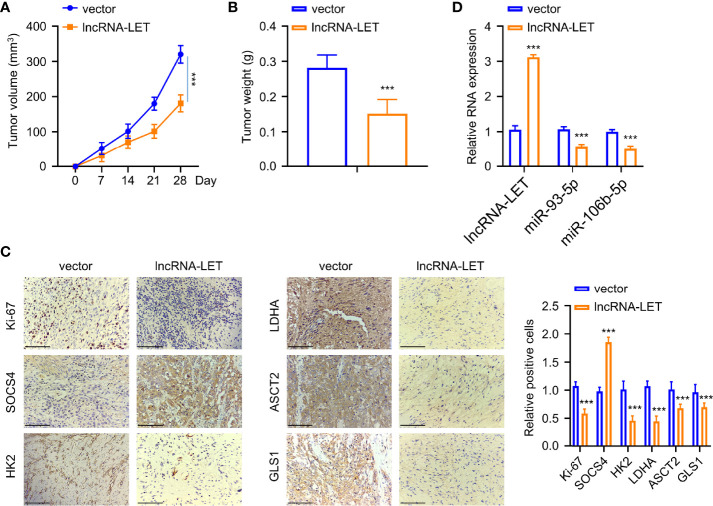
An increase of lncRNA-LET inhibited esophageal squamous cell carcinoma cell proliferation *in vivo*. **(A)** The subcutaneous tumor volume in the tumor xenograft model. **(B)** The tumor weights in different groups. **(C)** The expression of Ki-67, SOCS4, hK2, ldhA, ASCT2, and gls1 proteins in subcutaneous tumor tissues was detected by immunohistochemistry. **(D)** lncRNA-LET, miR-106b-5p, and miR-93-5p expression within subcutaneous tumor tissues were detected through qRT-PCR. *P < 0.05, **P < 0.01, and ***P < 0.001.

Finally, the Eca-109 tumor mice model was established for evaluating the influence of lncRNA-LET levels *in vivo*. Eca-109 cells transfected with or without lncRNA-LET were given into the dorsal side of nude mice *via* subcutaneous injection. As observed, the mice in the lncRNA-LET transfected group had a remarkably decreased tumor volume than those in the vector treatment group (**Figure 7A**). Correspondingly, the subcutaneous tumor weight in the lncRNA-LET-overexpression treatment group was lighter than those in the normal vector group (**Figure 7B**). The tumor tissues in the lncRNA-LET-overexpressed treatment group showed a lesser Ki-67 (proliferative marker) positive rate than the control vector group (**Figure 7C**). Notably, all cell metabolic markers (HK2, ldhA, ASCT2, and gls1) were downregulated in the lncRNA-LET treatment group. Contrarily, the positive trend of SOCS4 was observed in the lncRNA-LET tumor model, which was consistent with the positive correlation between lncRNA-LET and SOCS4. Finally, the miR-106b-5p and miR-93-5p within subcutaneous tumors of different groups were determined, indicating that their expression decreased in the lncRNA-LET group (**Figure 7D**).

## Discussion

ESCC is one of the life-threatening malignant tumors affecting numerous patients globally, requiring the exploration of the underlying mechanisms and subsequent development of treatment options. In this vein, the lncRNAs could regulate cancer progression through interacting with biological factors (24–26). Initially, we observed that lncRNA-LET was expressed lower in the ESCC cancer tissues compared to the paracancerous tissues. With the lncRNA-LET pcDNA transfection, the lncRNA-LET level was dramatically elevated, giving rise to the markedly reduced cell viability, which suggested the tumor inhibition function of lncRNA-LET. Moreover, it should be noted that the lncRNAs were involved in various metabolic processes (27). Thus, the levels of various metabolic substances reflected that overall glycolytic flux and mitochondrial oxidative respiration were obtained. As shown in **Figure 2**, data on the levels of glucose, lactate, and ATP indicated augmented lncRNA-LET levels, reducing intracellular glycolysis. Furthermore, the expressions of HK2 and LDHA in ESCC cells with the transfection of lncRNA-LET were measured *via* the Western blotting method. The HK2 and LDHA levels dramatically decreased due to lncRNA-LET overexpression. We further explore the roles of lncRNA-LET on metabolic transporters (ASCT2) responsible for glutamine transportation and enzymes (GLS1) involved in the TCA cycle *via* converting glutamate into α-KG (23, 28). We altogether firmly believe that lncRNA-LET played a critical role in cell glycolysis and the TCA cycle.

To this end, miRNAs play important roles in various cancers, exhibiting tumor-promoting or tumor-inhibiting effects (29–32). In this context, the expressions of miR-106b-5p and miR-93-5p were abnormal within ESCC tissues. The cell viability assay revealed that miR-106b-5p and miR-93-5p showed tumor promotion efficiency. In addition, the overexpression of these two miRNAs significantly affected the glucose uptake, ATP generation, and lactate generation, indicating their roles in regulating the metabolism in Eca-109 and KYSE410 cells. In addition, a reduction in HK2 and ldhA protein expressions in cells transfected with a single lncRNA-LET plasmid was observed. However, increased HK2 and ldhA expressions were observed in the co-transfection treatment group.

In humans, the SOCS family affects the release of cytokines and the downregulation of SOCS activity, resulting in excessive cytokine generation and promoting cancer development (24). Despite their importance, only a few reports concerning SOCS4 have been published to date (33). Subsequently, herein, we observed that miR-106b-5p and miR-93-5p harbored the binding sites of SOCS4. The tumor inhibitory roles of SOCS4 similar to lncRNA-LET were also demonstrated with functional and metabolic assays. Moreover, miR-106b-5p and miR-93-5p affected the SOCS4 levels, inducing a decrease of SOCS4 and reversing the functions of miR-106b-5p and miR-93-5p in cancer progression.

As lncRNA-LET was downregulated within ESCC cells and tissues, we increased the lncRNA-LET levels, in which inhibition of the proliferation, glycolysis, and glutamine decomposition of ESCC cells was observed. Subsequently, it was revealed that lncRNA-LET targeted miR-106b-5p and miR-93-5p, affecting glycolysis and the glutamine decomposition of ESCC cells. As the SOCS4 proteins are downstream targets of both miRNAs, the overexpression of SOCS4 correspondingly inhibited the glycolysis and glutamine decomposition of ESCC cells. In addition, lncRNA-LET overexpression inhibited ESCC cell proliferation *in vivo*. The interactions of lncRNA-LET, miR-106b-5p, miR-93-5p, and SOCS4 were altogether investigated, and hopefully, this axis could be a promising target for ESCC.

## Conclusion

In summary, our findings revealed the role of lncRNA-LET in repressing tumor cell survival *in vitro* and *in vivo*. In addition, lncRNA-LET could affect miR-106b-5p and miR-93-5p, while SOCS4 affected cell metabolism, thus inhibiting tumor progression. Our results altogether indicated that lncRNA-LET, miR-106b-5p, or the miR-93-5p–SOCS4 axis might contribute to ESCC treatment.

## Data Availability Statement

The raw data supporting the conclusions of this article will be made available by the authors without undue reservation.

## Ethics Statement

The studies involving human participants were reviewed and approved by the medical ethics committee of the 2nd Affiliated Hospital of FMU. The patients/participants provided their written informed consent to participate in this study. The animal study was reviewed and approved by the medical ethics committee of the 2nd Affiliated Hospital of FMU.

## Author Contributions

ZC, ZH, and JL contributed to the conception of the study. XSu and CXu performed the data analyses and wrote the manuscript. CXi and XSi performed the research and collected data. JX and WH helped performed the analysis with constructive discussions. All authors contributed to the article and approved the submitted version.

## Funding

This study was supported by the Natural Science Foundation of Fujian Province (2019J01476 and 2021J01273), the Medical Innovation Foundation of Fujian (2019-CXB-19), and Quanzhou High-Level Talent Plan (2020C003R).

## Conflict of Interest

The authors declare that the research was conducted in the absence of any commercial or financial relationships that could be construed as a potential conflict of interest.

## Publisher’s Note

All claims expressed in this article are solely those of the authors and do not necessarily represent those of their affiliated organizations, or those of the publisher, the editors and the reviewers. Any product that may be evaluated in this article, or claim that may be made by its manufacturer, is not guaranteed or endorsed by the publisher.
