# Supplementary figures and images for: lncRNA-LET Regulates Glycolysis and Glutamine Decomposition of Esophageal Squamous Cell Carcinoma Through miR-93-5p/miR-106b-5p/SOCS4
Source: Front Oncol. 2022 May 10;12:897751. doi: 10.3389/fonc.2022.897751 (PMC9127425; doi:10.3389/fonc.2022.897751)

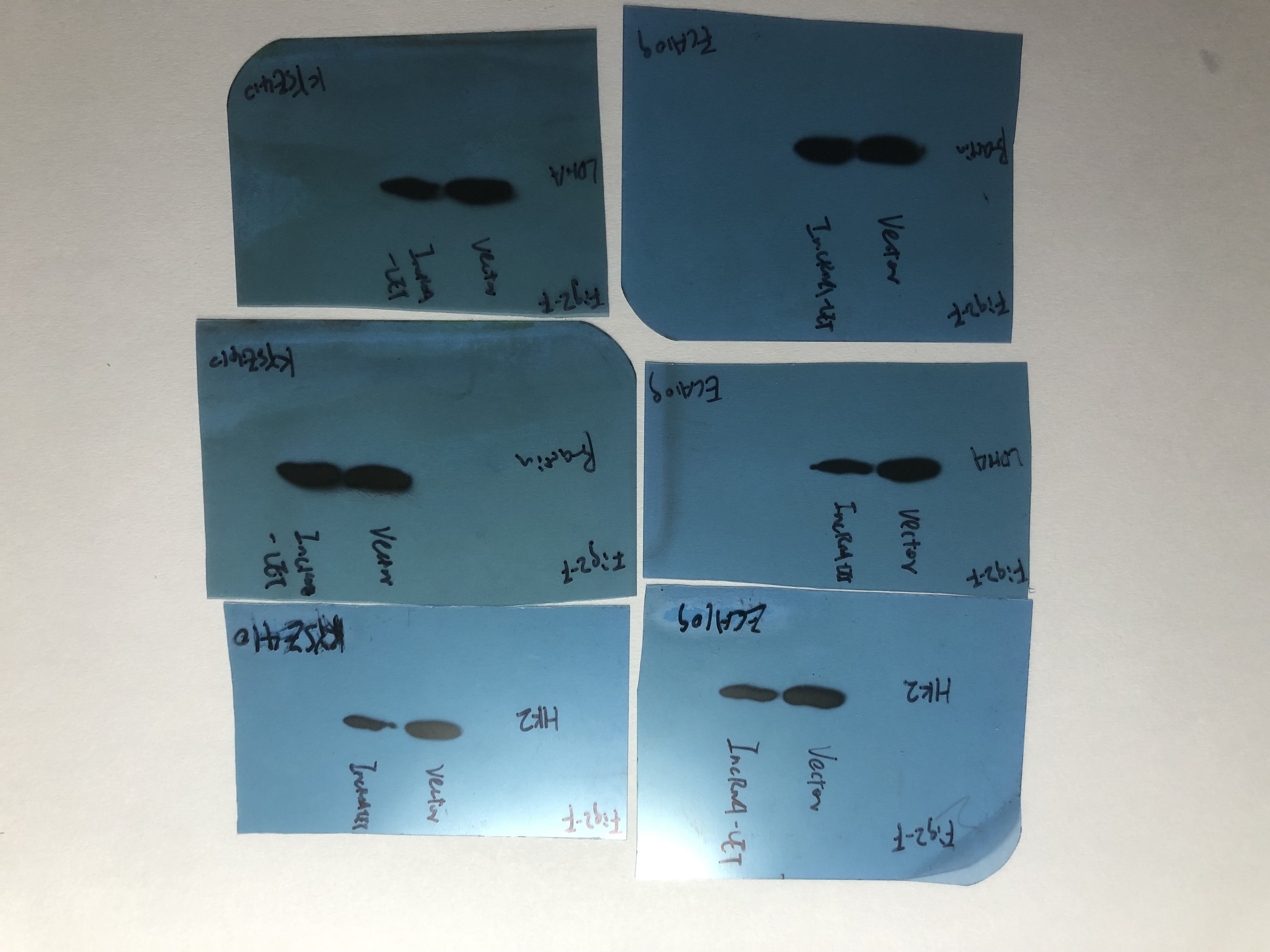

Supplement: Supplementary file 1 [file Image_1.jpeg]

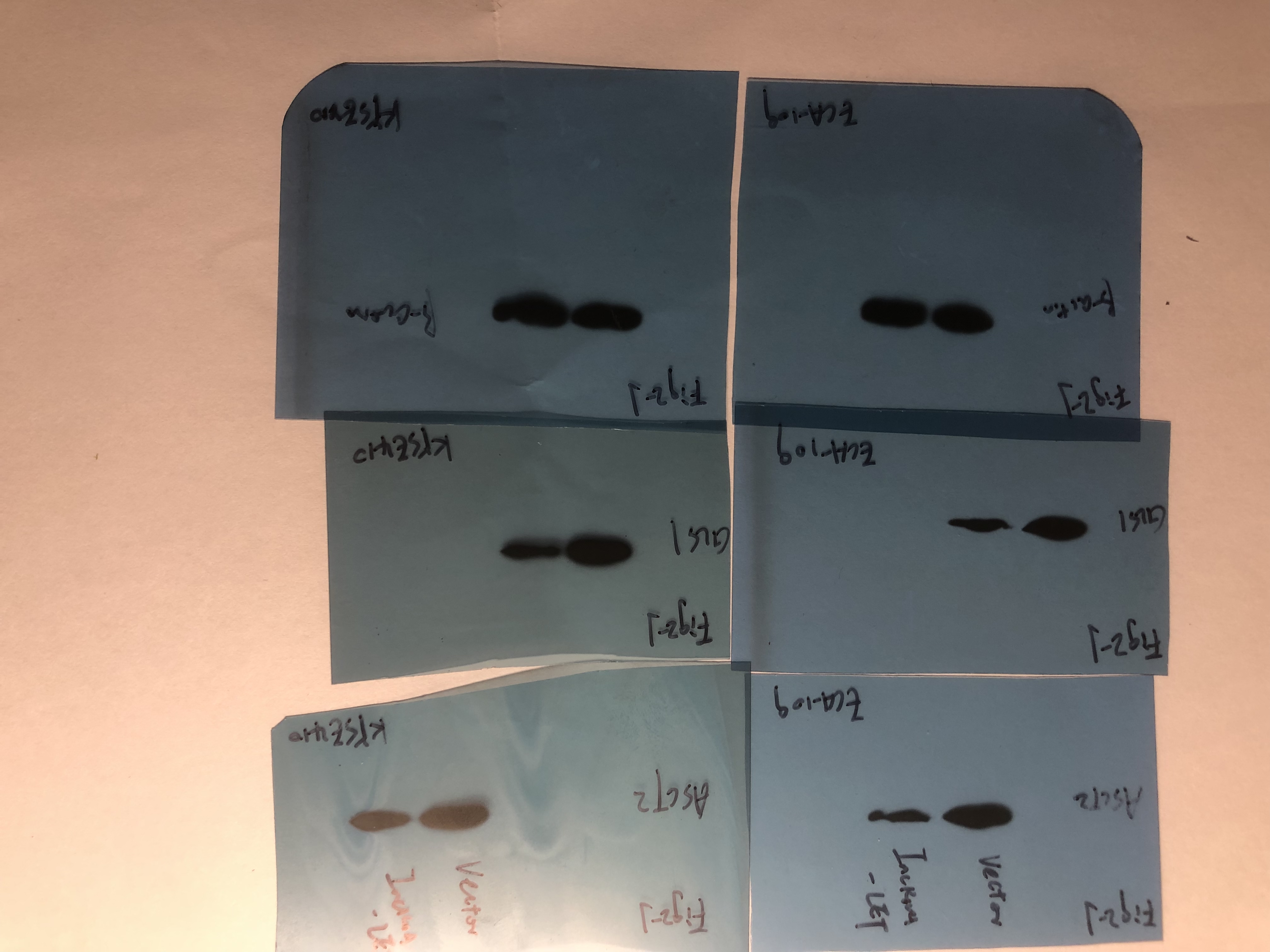

Supplement: Supplementary file 2 [file Image_2.jpeg]

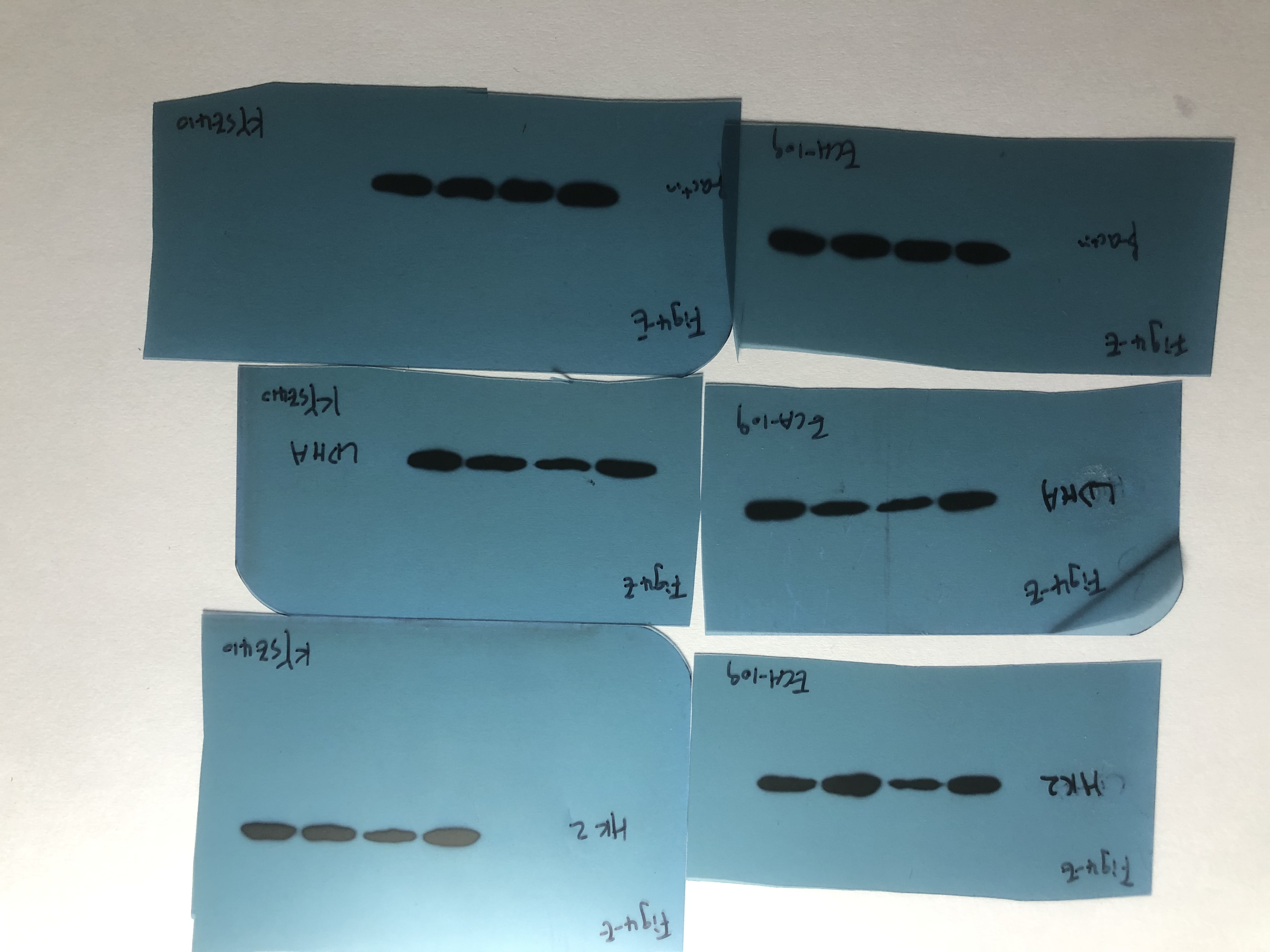

Supplement: Supplementary file 3 [file Image_3.jpeg]

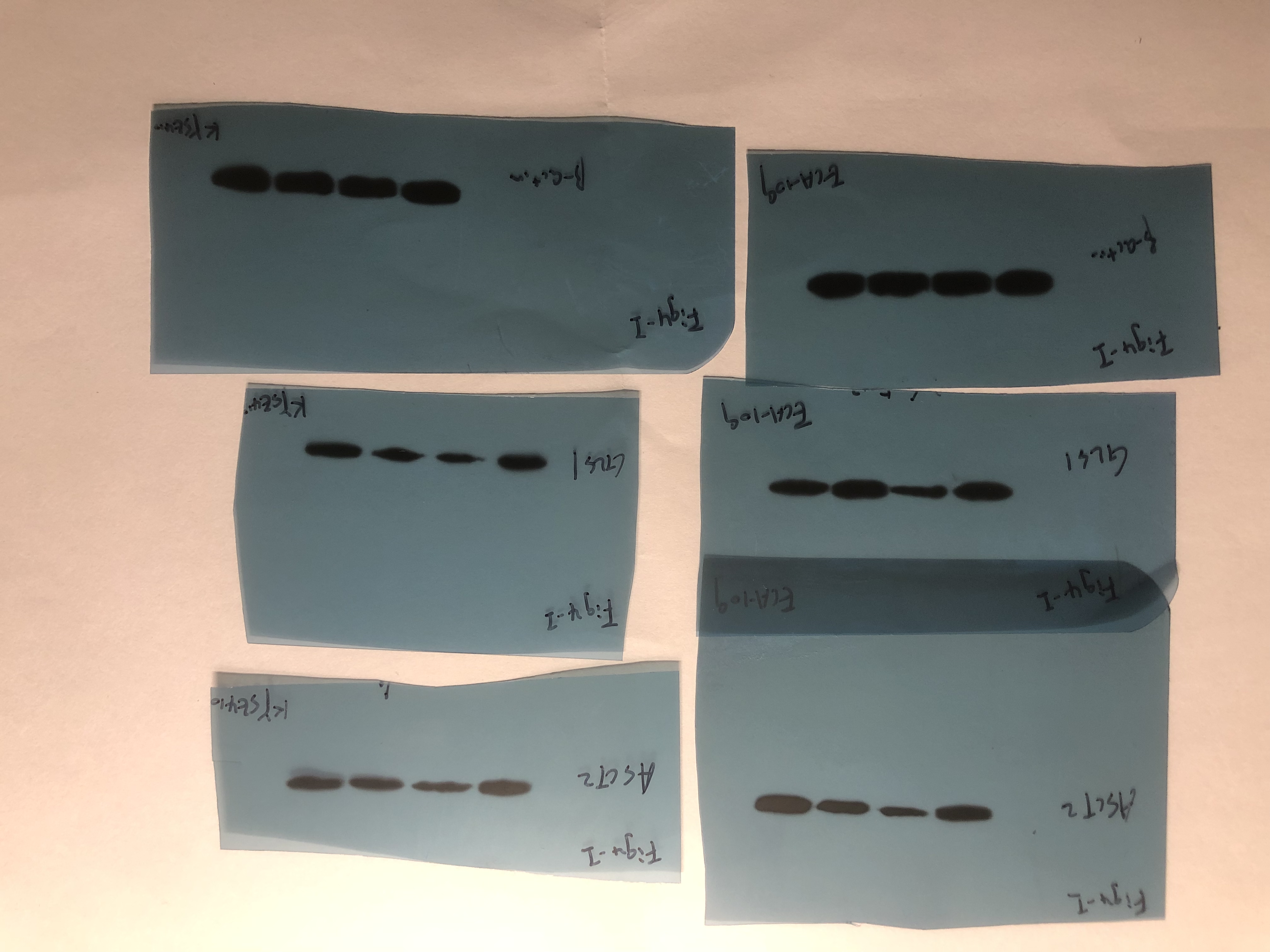

Supplement: Supplementary file 4 [file Image_4.jpeg]

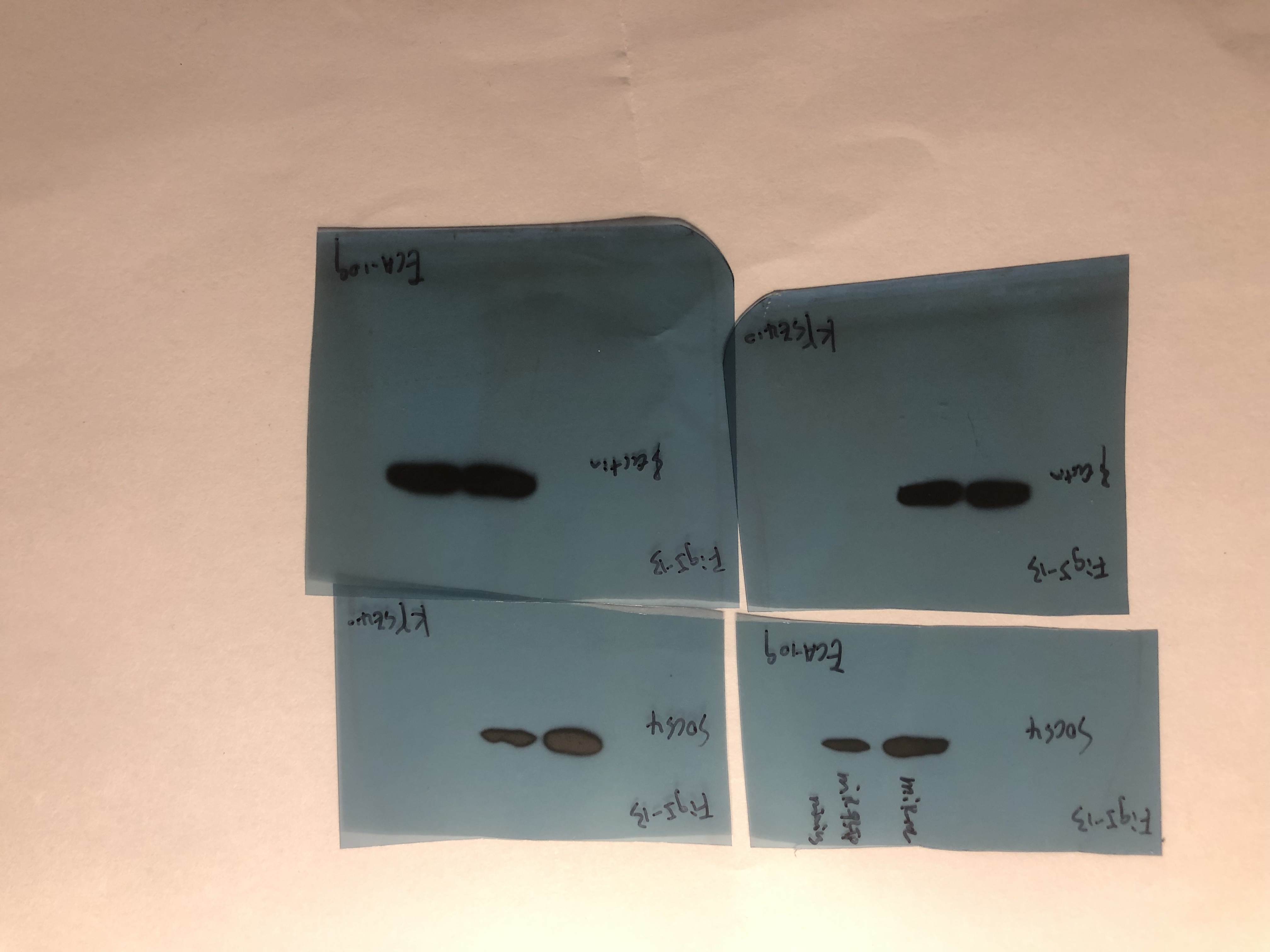

Supplement: Supplementary file 5 [file Image_5.jpeg]

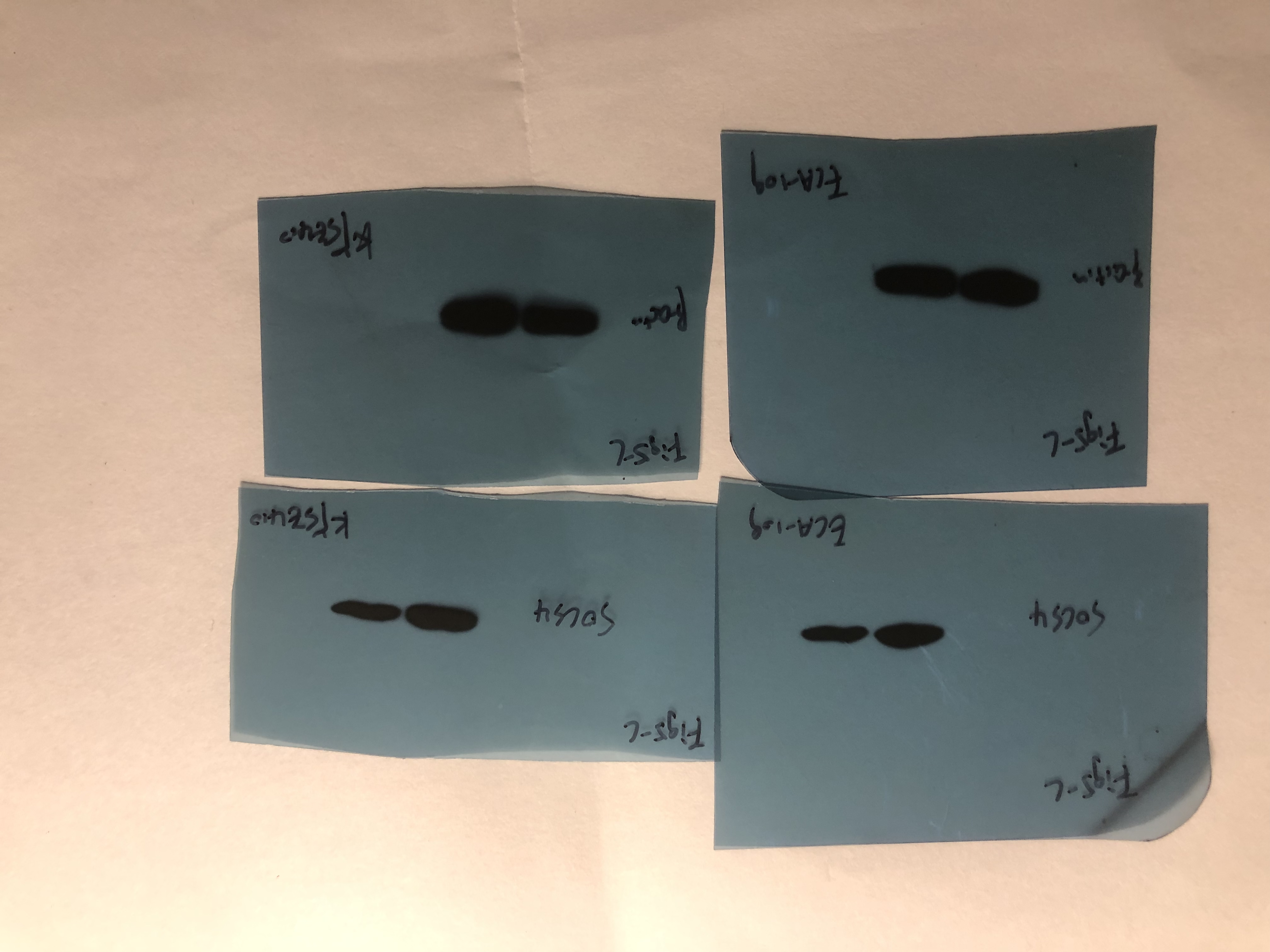

Supplement: Supplementary file 6 [file Image_6.jpeg]

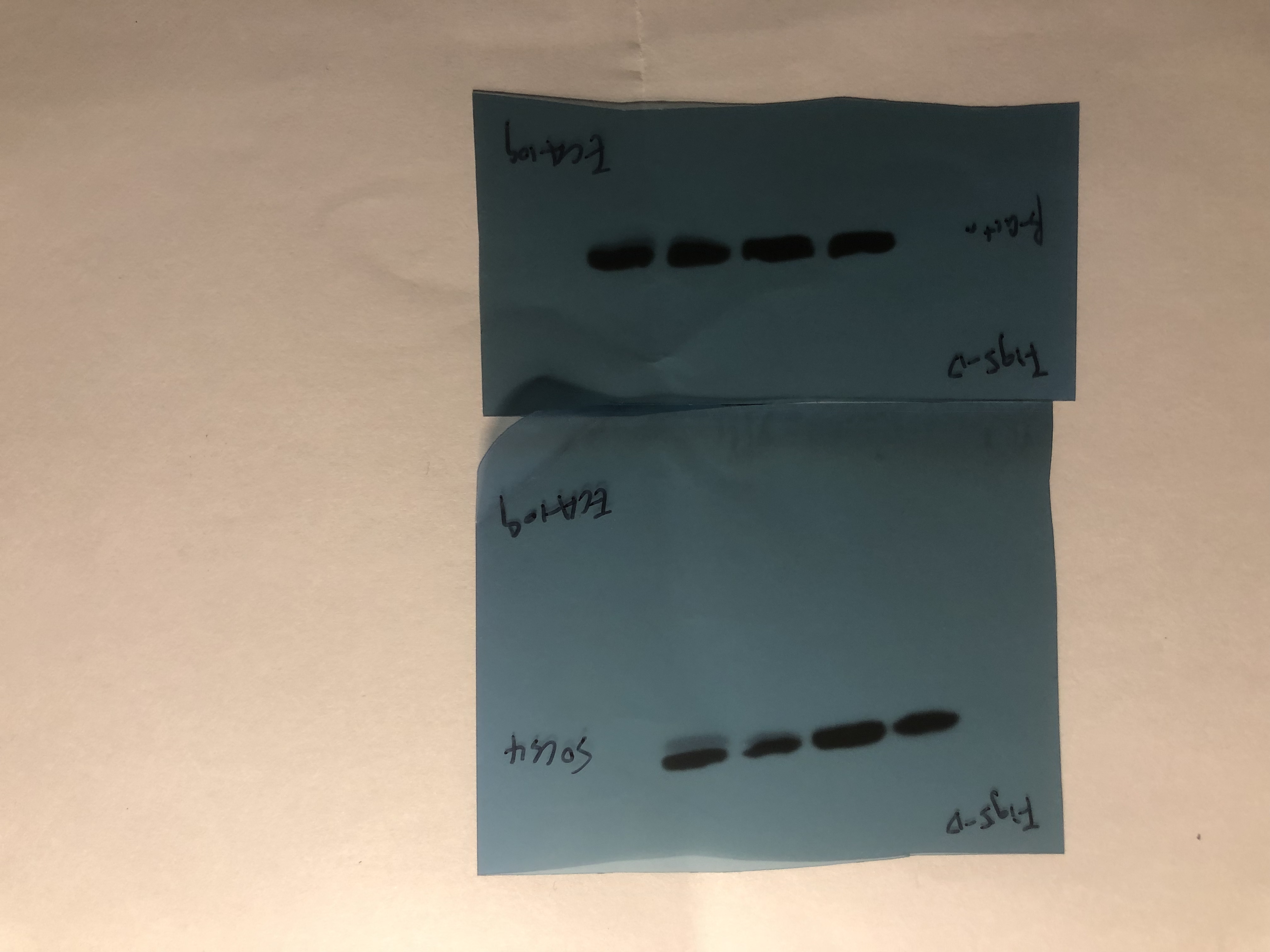

Supplement: Supplementary file 7 [file Image_7.jpeg]

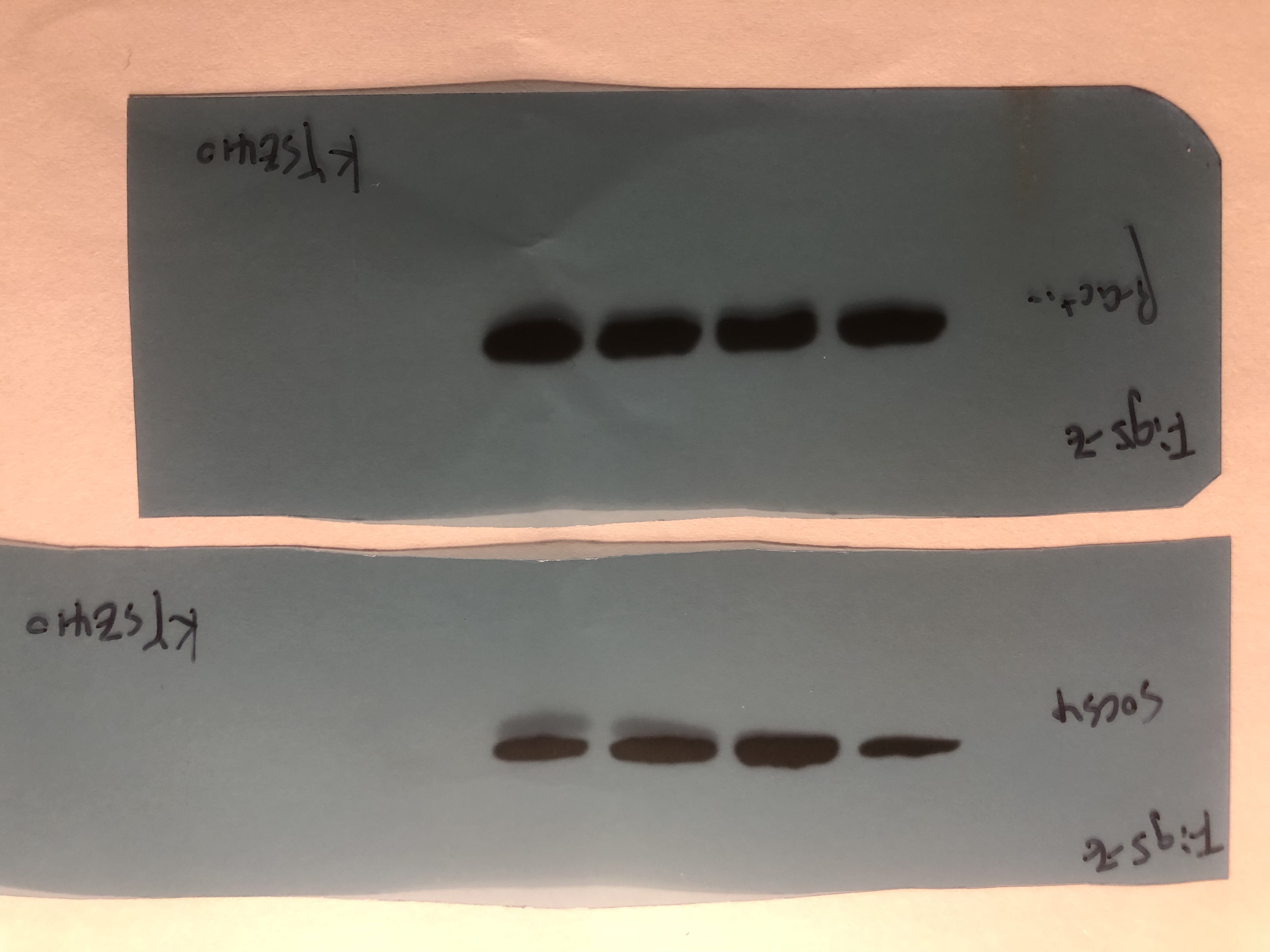

Supplement: Supplementary file 8 [file Image_8.jpeg]

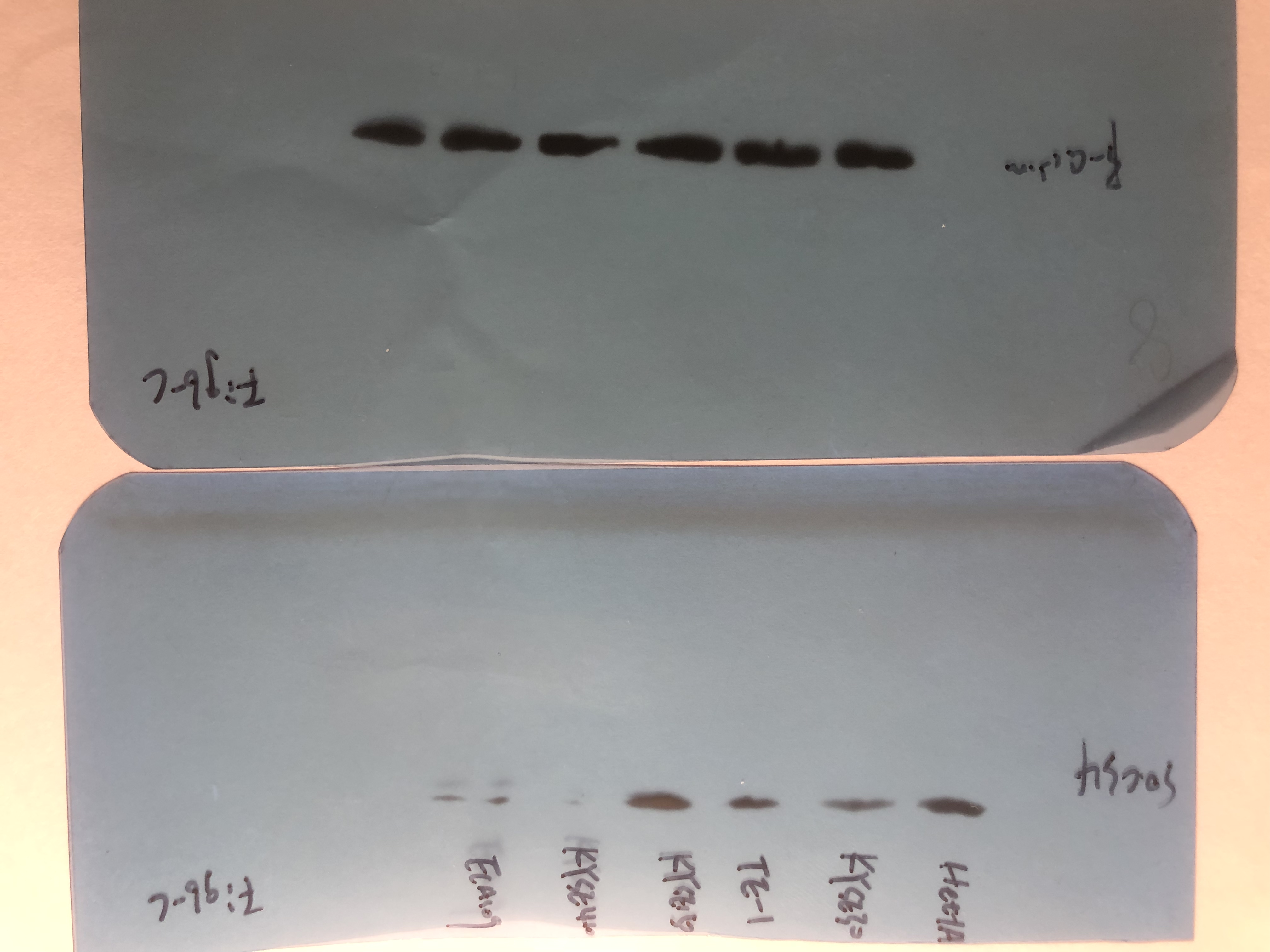

Supplement: Supplementary file 9 [file Image_9.jpeg]

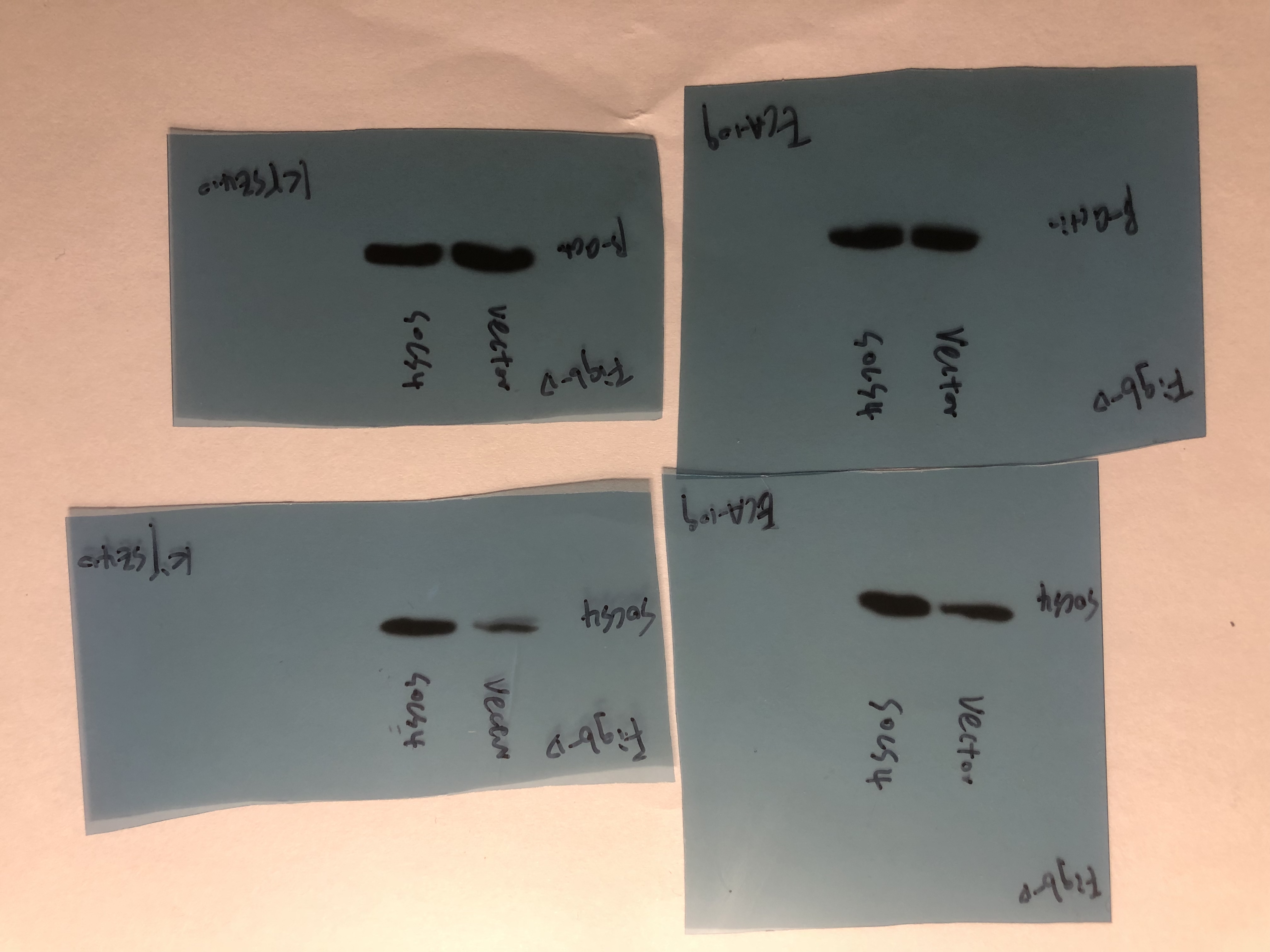

Supplement: Supplementary file 10 [file Image_10.jpeg]

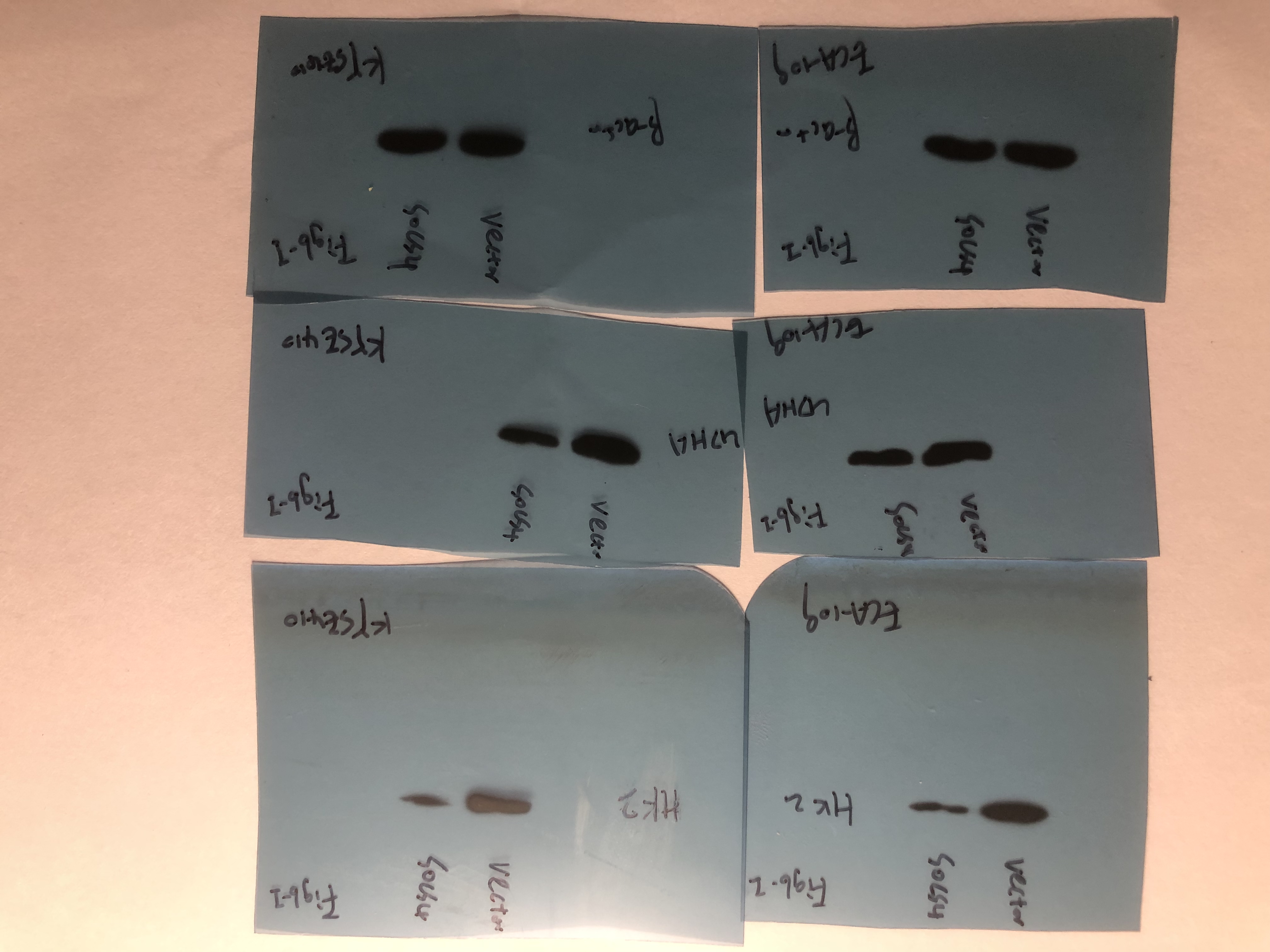

Supplement: Supplementary file 11 [file Image_11.jpeg]

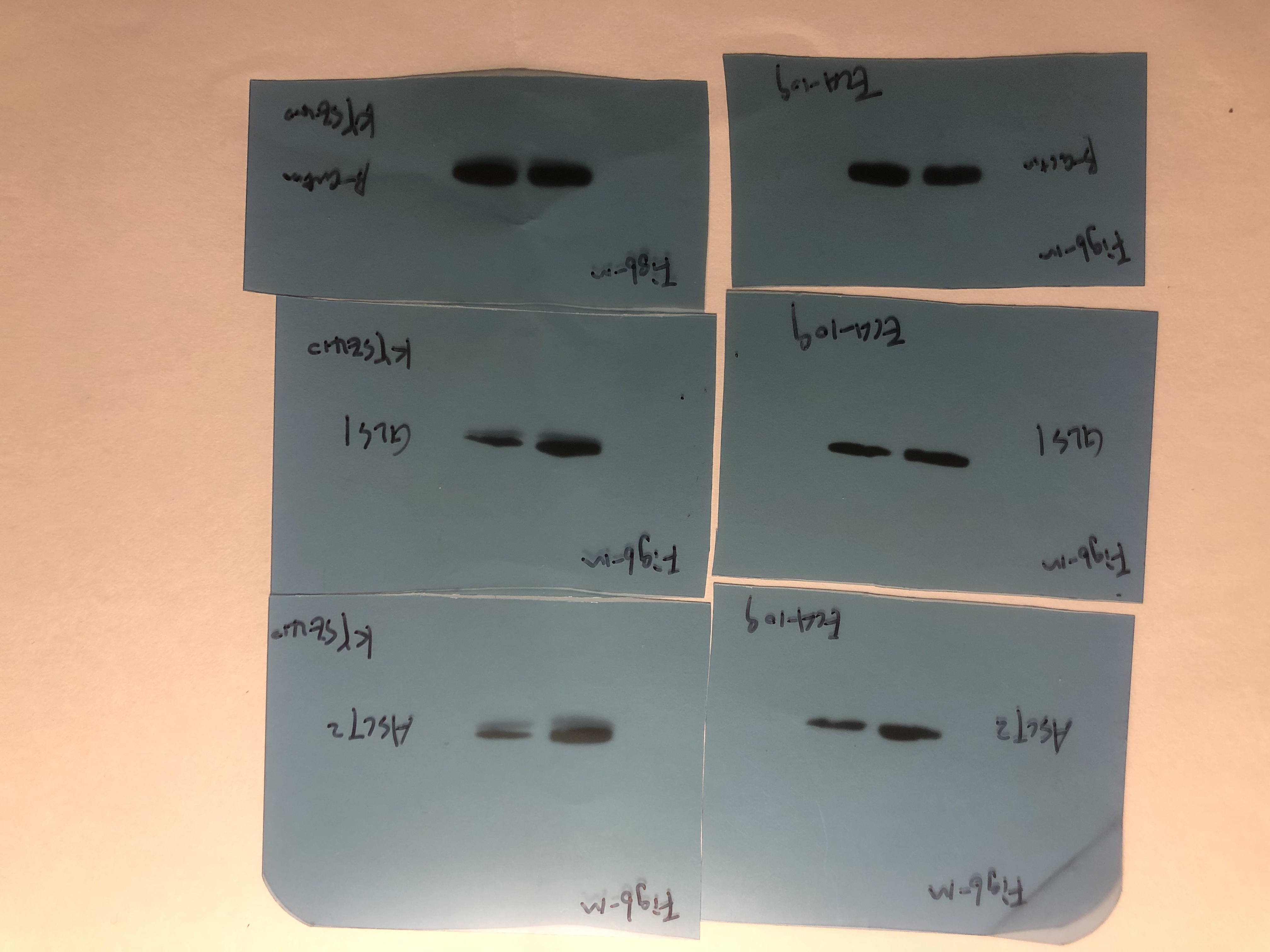

Supplement: Supplementary file 12 [file Image_12.jpeg]
